# Lipoproteins Are Responsible for the Pro-Inflammatory Property of *Staphylococcus aureus* Extracellular Vesicles

**DOI:** 10.3390/ijms22137099

**Published:** 2021-07-01

**Authors:** Pradeep Kumar Kopparapu, Meghshree Deshmukh, Zhicheng Hu, Majd Mohammad, Marco Maugeri, Friedrich Götz, Hadi Valadi, Tao Jin

**Affiliations:** 1 Department of Rheumatology and Inflammation Research, Institute of Medicine, Sahlgrenska Academy, University of Gothenburg, 41346 Gothenburg, Sweden; meghshreed@gmail.com (M.D.); zhicheng.hu@gu.se (Z.H.); majd.mohammad@rheuma.gu.se (M.M.); mgrmarco@gmail.com (M.M.); hadi.valadi@gu.se (H.V.); tao.jin@rheuma.gu.se (T.J.); 2 Center for Clinical Laboratories, The Affiliated Hospital of Guizhou Medical University, Guiyang 550001, China; 3 Department of Microbial Genetics, Interfaculty Institute of Microbiology and Infection Medicine Tübingen (IMIT), University of Tübingen, 72076 Tübingen, Germany; friedrich.goetz@uni-tuebingen.de; 4 Department of Rheumatology, Sahlgrenska University Hospital, 41345 Gothenburg, Sweden

**Keywords:** *Staphylococcus aureus*, extracellular vesicles, lipoproteins, inflammation, TLR2

## Abstract

*
Staphylococcal aureus* (*S. aureus*), a Gram-positive bacteria, is known to cause various infections. Extracellular vesicles (EVs) are a heterogeneous array of membranous structures secreted by cells from all three domains of life, i.e., eukaryotes, bacteria, and archaea. Bacterial EVs are implied to be involved in both bacteria–bacteria and bacteria–host interactions during infections. It is still unclear how *S. aureus* EVs interact with host cells and induce inflammatory responses. In this study, EVs were isolated from *S. aureus* and mutant strains deficient in either prelipoprotein lipidation (Δ*lgt*) or major surface proteins (Δ*srtAB*). Their immunostimulatory capacities were assessed both in vitro and in vivo. We found that *S. aureus* EVs induced pro-inflammatory responses both in vitro and in vivo. However, this activity was dependent on lipidated lipoproteins (Lpp), since EVs isolated from the Δ*lgt* showed no stimulation. On the other hand, EVs isolated from the Δ*srtAB* mutant showed full immune stimulation, indicating the cell wall anchoring of surface proteins did not play a role in immune stimulation. The immune stimulation of *S. aureus* EVs was mediated mainly by monocytes/macrophages and was TLR2 dependent. In this study, we demonstrated that not only free Lpp but also EV-imbedded Lpp had high pro-inflammatory activity.

## 1. Introduction

Extracellular vesicles (EVs) are a heterogeneous array of membranous structures secreted by cells from all three domains of life, i.e., eukaryotes [1,2,3,4,5,6], bacteria [7], and archaea [3]. The physical size of EVs can vary significantly depending on their cell origin and EV subtypes. Currently, operational terms are used to define EV types as well as specific methods used for isolation of subpopulations; examples of size designations used are “small EVs” (<100 nm or <200 nm in diameter) and large and/or medium EVs (>200 nm) [8]. In addition, there are operational terms to further define EV types, e.g., EVs derived from endosomes are termed exosomes (EVs containing endosome markers), and plasma membrane-derived EVs are referred to as ectosomes (microparticles/microvesicles) [8,9,10]. Nevertheless, EVs facilitate intercellular interaction that enables the cell-to-cell transfer of lipids, proteins, genetic cargo, and metabolites to distant recipient cells [11,12]. Recent studies have found that EVs play a crucial role in different diseases including cancer [1,13], neurodegenerative disorders [14], heart disorders [15,16], pulmonary disorders [17], and renal disorders [18,19].

Bacterial EVs play a role in the pathophysiology of bacteria–bacteria and bacteria–host interactions through cell–vesicle communication that involves the transport of various components to recipient cells, including virulence factors and toxins that can modulate host immunity [20]. Gram-negative bacteria-derived EVs are primarily derived from the outer membrane and are referred to as outer membrane vesicles (OMV), and are composed of proteins [21,22], lipids [23], and nucleic acids [24]. They facilitate bacteria–bacteria interactions that enable the transfer of components and the formation of biofilms, whereas bacteria–host interactions elicit a variety of responses, ranging from non-immunogenic to pro-inflammatory reactions or cytotoxicity, depending on the type of target cells [20,25,26].

Gram-positive bacteria also secrete EVs, but there have been fewer studies in the field of intercellular vesicle transport to date. *Staphylococcus aureus* (*S. aureus*) is a Gram-positive bacteria causing a wide range of diseases, from skin infections and food poisoning to life-threatening conditions such as sepsis, meningitis, and endocarditis. *S. aureus* infectious diseases are triggered by a variety of virulence factors, including surface proteins, cell wall components, toxins, and enzymes [27,28,29,30,31]. Recent shreds of evidence show that many *S. aureus* components can also be released as part of EVs [32]. Well-known virulence proteins such as coagulases, hemolysin, and IgG-binding protein SbI are among the 90 vesicular proteins found in *S. aureus* [32]. *S. aureus* EVs have been detected in skin samples from atopic dermatitis (AD) patients and are associated with AD development [33], whereas in vitro *S. aureus* EVs exert cytotoxic responses in a dose-dependent manner [34]. In a very recent study, *S. aureus* EVs have been highlighted as having a potential role in the application of vaccine platforms [35]. However, bacterial EVs are known to induce pro-inflammatory responses [36], which may cause unwanted side effects in vaccination.

So far it is still largely unknown which molecules are responsible for the immune-stimulating effect of *S. aureus* EVs. Here, we demonstrate that lipoproteins (Lpps) determined the pro-inflammatory capacity of *S. aureus* EVs in both in vitro and in vivo settings through Toll-like receptor 2 (TLR2) dependent mechanisms.

## 2. Results

### 2.1. In vitro Stimulation of S. aureus EVs Causes a Pro-Inflammatory Response

**Figure 1:**
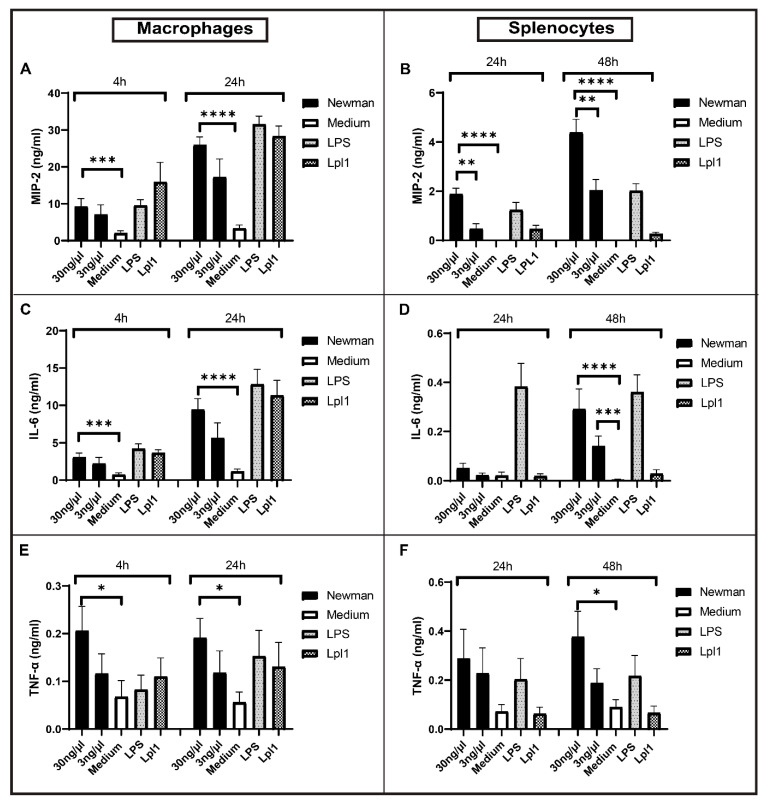
In Vitro stimulation of *S. aureus* Newman EVs in murine peritoneal macrophages and splenocytes. (**A**,**C**,**E**) MIP-2, IL-6, and TNF-α stimulation in peritoneal macrophages; (**B**,**D**,**F**) MIP-2, IL-6, and TNF-α stimulation in splenocytes. Statistical analyses were performed using the Mann–Whitney U test and the data represented as the mean with SEM * *p* < 0.05; ** *p* < 0.01; *** *p* < 0.001; **** *p* < 0.0001.

To assess the immune-stimulating effect of *S. aureus* EVs, mouse peritoneal macrophages and splenocytes were stimulated with EVs isolated from the *S. aureus* wild-type strain Newman. LPS and Lpl1 (lipoprotein) were used as positive controls while the medium served as a negative control. Significantly elevated levels of MIP-2 were found when peritoneal macrophages were stimulated with Newman EVs at both 4 and 24 h time points (Figure 1A), and similar results were found in splenocytes stimulated at 24 and 48 h (Figure 1B). Additionally, there was a significant dose-dependent manner of the stimulation of splenocytes at both 24 and 48 h time points (Figure 1B), while no differences in peritoneal macrophages were observed (Figure 1A). IL-6 also showed a significant increase upon stimulation of EVs at 4 and 24 h in peritoneal macrophages (Figure 1C) and 48 h in splenocytes (Figure 1D). Similarly, TNF-α production was increased at 4 and 24 h in peritoneal macrophages (Figure 1E) and 48 h in splenocytes (Figure 1F). Strikingly, *S. aureus* EVs induced comparable or even higher levels of certain chemokines/cytokines (MIP-2 and TNF-α) than the positive controls (LPS and lipoproteins, Lpl1). Collectively, these data suggest that EVs from *S. aureus* contain components that caused pro-inflammatory reactions in host cells.

### 2.2. The Pro-Inflammatory Property of S. aureus EVs Is Dependent on TLR2 Signaling

**Figure 2:**
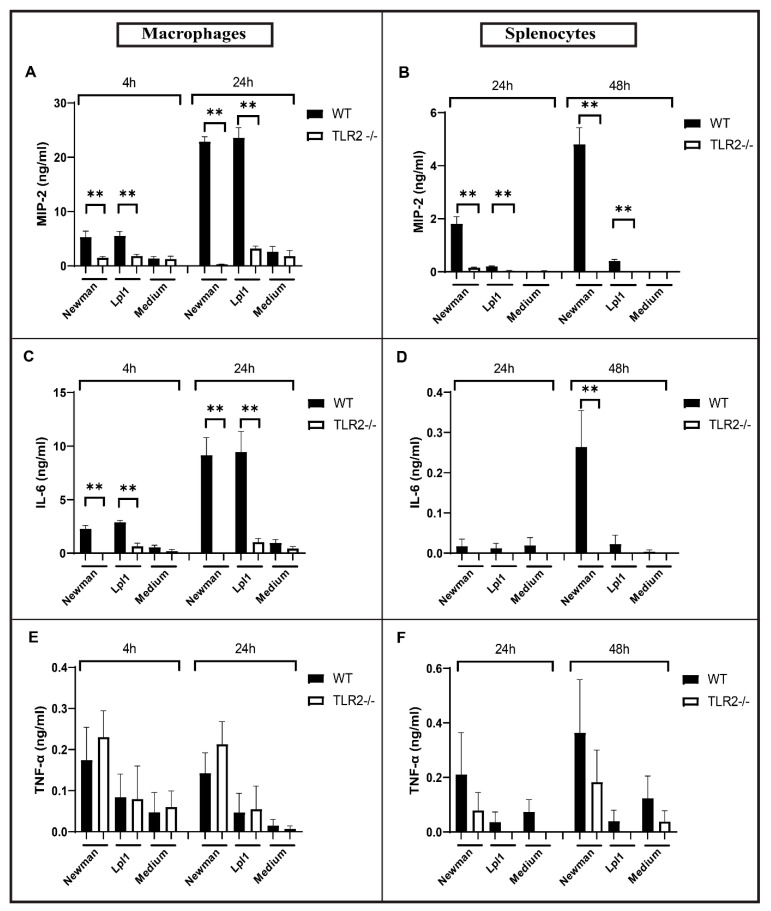
In Vitro stimulation of *S. aureus* EVs in the peritoneal macrophages and splenocytes of wild-type (WT) and Toll-like receptor 2 deficient mice (TLR2^−/−^). (**A**,**C**,**E**) MIP-2, IL-6, and TNF-α stimulation in peritoneal macrophages; (**B**,**D**,**F**) MIP-2, IL-6, and TNF-α stimulation in splenocytes. Statistical analyses were performed using the Mann–Whitney U test and the data represented as the mean with SEM ** *p* < 0.01.

To verify whether the immune stimulatory effects of *S. aureus* EVs were dependent on TLR2 signaling, peritoneal macrophages, and splenocytes from TLR2^−/−^ and C57Bl/6 wild-type mice were stimulated with EVs from the Newman strain. MIP-2 levels were significantly reduced to the levels of non-stimulated cells for all the time points in the peritoneal macrophages (Figure 2A) and splenocytes (Figure 2B) isolated from TLR2^−/−^ mice, compared with C57Bl/6 wild-type mice. Similarly, IL-6 release by macrophages and splenocytes upon EVs stimulation was regulated by TLR2 (Figure 2C,D). There were no significant differences in TNF-α production when cells from TLR2^−/−^ and C57Bl/6 wild-type mice were stimulated with Newman EVs (Figure 2E,F). Our data suggest that the pro-inflammatory response induced by EVs was largely dependent on the TLR2 signaling pathway.

### 2.3. Lipoproteins Determine the Pro-Inflammatory Property of S. aureus EVs

**Figure 3:**
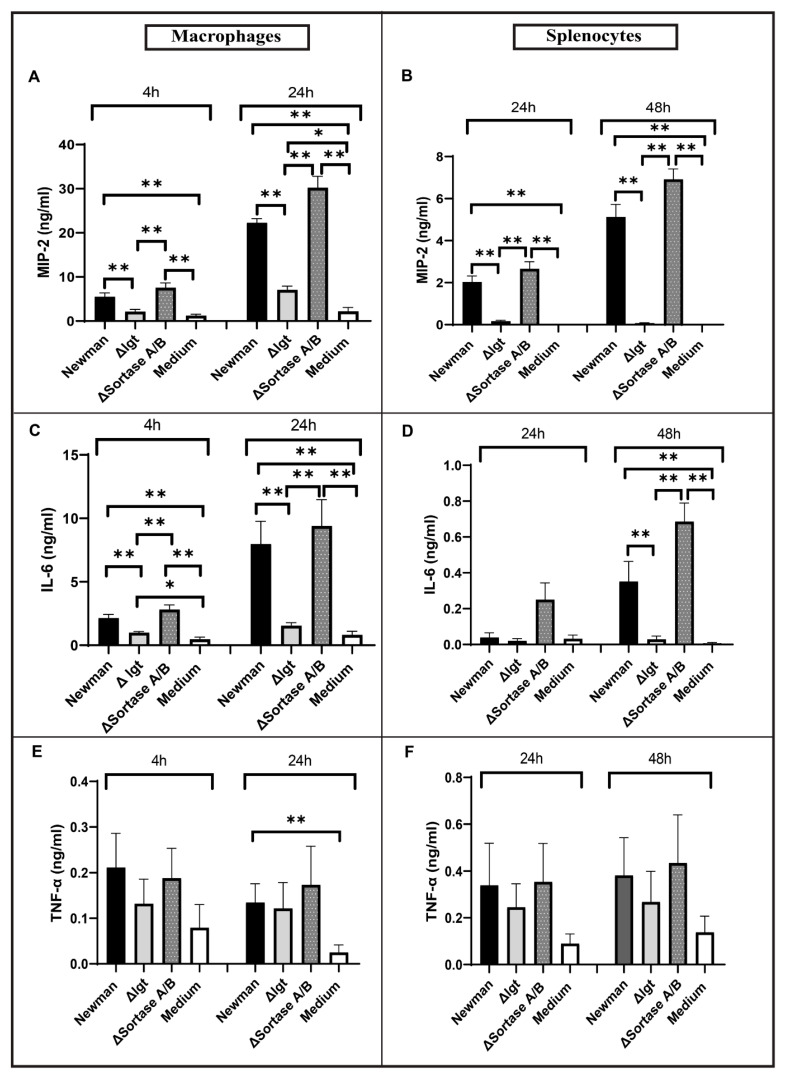
Mouse peritoneal macrophages and splenocytes stimulated with *S. aureus* EVs derived from Newman, Newman Δ*lgt*, and Newman Δ*srtAB*. (**A**,**C**,**E**) MIP-2, IL-6, and TNF-α stimulation in peritoneal macrophages; (**B**,**D**,**F**) MIP-2, IL-6, and TNF-α stimulation in splenocytes. Statistical analyses were performed using the Mann–Whitney U test and the data represented as the mean with SEM ** *p* < 0.01.

*S. aureus* Lpps were shown to be among the most potent components in *S. aureus* inducing inflammation via TLR2 [28,37]. As cytokine release induced by *S. aureus* EVs is TLR2 dependent, we hypothesized that Lpps are the major component responsible for the pro-inflammatory property of EVs secreted from *S. aureus*. To verify this hypothesis, peritoneal macrophages and splenocytes of mice were stimulated with the *S. aureus* EVs derived from the bacterial strains wild-type Newman, Newman Δ*lgt*, and Newman Δ*srtAB*. As shown in Figure 3, the MIP-2 levels were significantly lower in EVs from Δ*lgt* compared with parental strain Newman at both time points in both peritoneal macrophages (Figure 3A) and splenocytes (Figure 3B). A similar pattern of stimulation response regarding IL-6 levels was observed at both time points in peritoneal macrophages (Figure 3C) and 48 h in splenocytes (Figure 3D, 48 h). In contrast, the EVs isolated from Newman Δ*srtAB* maintained their pro-inflammatory property (Figure 3). Notably, EVs from Δ*lgt* induced only slightly increased levels of MIP-2 at 24 h and IL-6 at 4 h in macrophage stimulation in contrast with the negative control (Figure 3A,C). Our data strongly suggested that Lpps are the major component responsible for the pro-inflammatory capacity of *S. aureus* EVs, but other molecules from EVs may also induce an immune response.

As observed in Figure 2, the elevated levels of MIP-2 and IL-6 in the Newman parental strain were TLR2-dependent. Therefore, we further studied the immune-stimulating capacities of EVs from different mutant strains in TLR2-deficient mice. EVs from Newman Δ*srtAB* possessed the same stimulatory capacity as the parental strain on immune cells isolated from C57Bl/6 wild-type mice but lost their immunogenicity on cells from TLR2^−/−^ mice (Supplementary Figure S1). In contrast, EVs from the Δ*lgt* mutant lacked immune-stimulating properties in cells from both C57Bl/6 wild-type mice and TLR2^−/−^ mice (Supplementary Figure S1).

### 2.4. Characterization of EVs from S. aureus Strains

**Figure 4:**
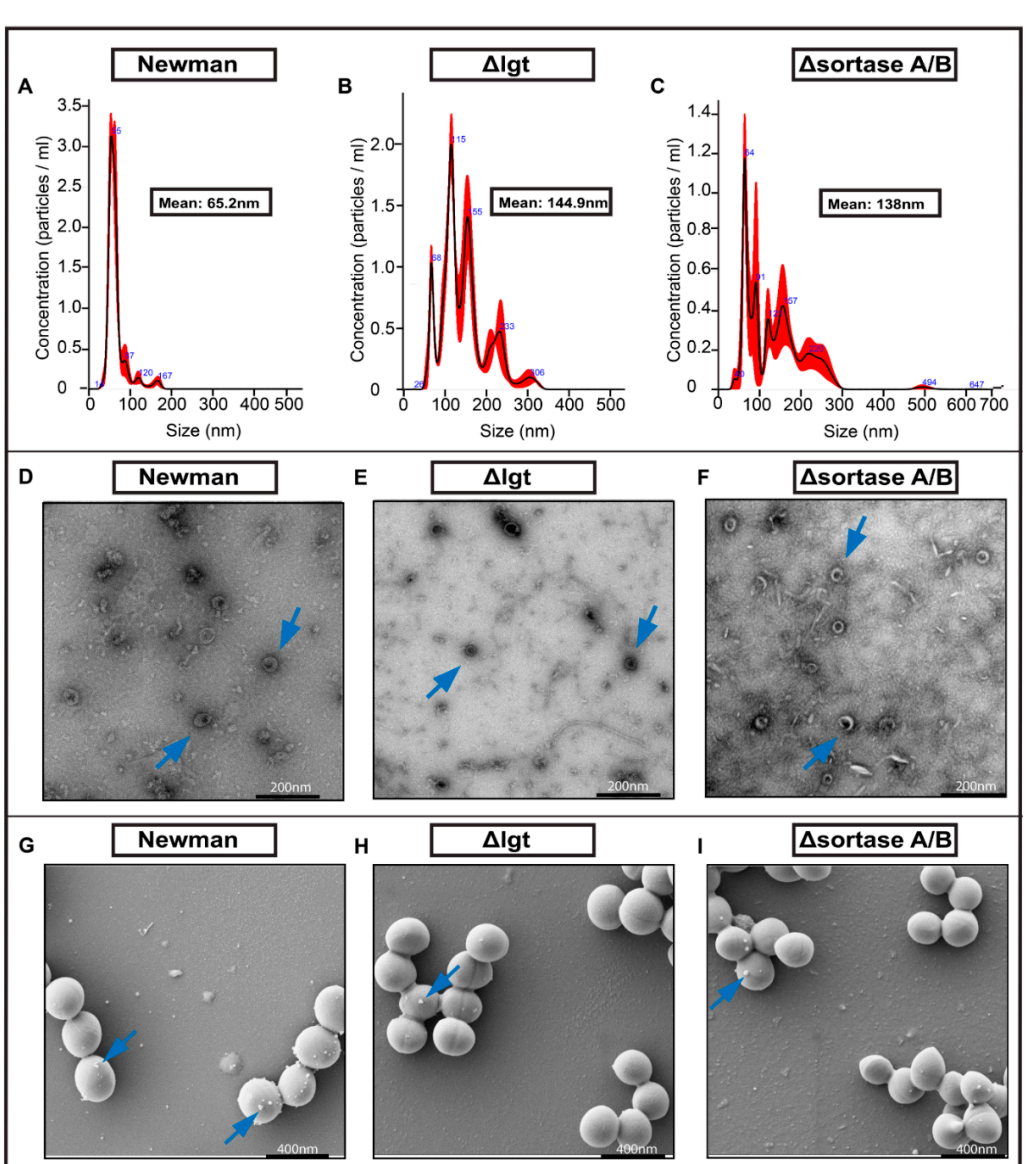
Characterization of extracellular vesicles (EVs). The size distribution of isolated EVs from Newman, Newman Δ*lgt*, Newman Δ*srtAB* (**A**–**C**). EVs were assessed for their size (nm) and concentration (particles/mL) using LM10 (Malvern Panalytical). Four independent measurements (biological replicates) were performed in scatter mode. Measurement readings for each EV sample were taken via five captures for 60 s each at 25 frames per second (fps), at adjusted camera levels (10–16) and detection thresholds (5–15) depending on the individual sample and manual monitoring of temperature. Electronic microscopy images of the isolated EVs (**D**–**F**) and presence of EVs in 3 h bacterial culture from Newman and mutant strains respectively (**G**–**I**). Arrow in (**D**–**F**) indicates EVs, while in the (**G**–**I**) images, budding-like dots indicate EVs on the cell surface of *S. aureus*.

The size and concentration of isolated EVs were determined using Nanosight tracking analysis (which is based on Brownian particle motion analyses as described in [38]) and transmission electron microscopy (TEM). Isolated EVs from all three bacterial strains used in this study had a heterogeneous size, ranging between 30 and 300 nm in diameter (Figure 4A–C). The mean size of EVs secreted from these three strains varied significantly. EVs from wild-type Newman cells had a mean size of 65.2 nm (Figure 4A), while the size of EVs from lipoprotein was deficient strain Δ*lgt* had a bigger variation with a mean size of 144.9 nm (Figure 4B). Similarly, the EVs from sortase A/B deficient strain Δ*srtAB* had a mean size of 138 nm (Figure 4C). This suggests that deficiency of *lgt* and *srtAB* had an impact on the size of EVs secreted by *S. aureus*. Subsequently, the morphology of isolated EVs was accessed by TEM. Figure 4D–F show TEM images of membranous vesicles, secreted by the bacterial strains Newman, Δ*lgt*, and Δ*srtAB* respectively with an approximate size of 300 nm in diameter. 3D analyses of the TEM images corroborated that these EVs are round structures with heterogeneous sizes (Figure 4D–F). Furthermore, we screened the *S. aureus* Newman and the respective mutant strains to check the budding of EVs from live bacteria with the help of SEM at two different time points, 3 h, and 24 h. The Newman strain released a bigger number of EVs compared with Δ*lgt* and Δ*srtAB* strains at 3 h of bacterial culture (Figure 4G–I and Supplementary Figure S2A). We also noticed that the production of EVs was less at 24 h in the bacterial culture samples than at 3 h (Supplementary Figures S2B and S3).

### 2.5. Lpps Are the Causatives for the Inflammation Induced by S. aureus EVs in Mice through Monocytes/Macrophages Mediated by TLR2

**Figure 5:**
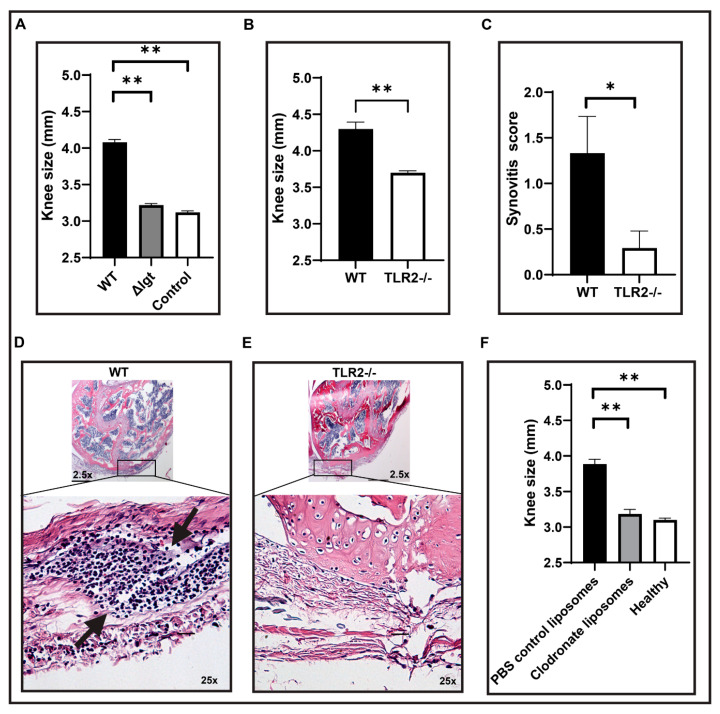
Lipoproteins in *S. aureus* EVs induce inflammation through monocytes/macrophages via TLR2 in mice. Size of the knee joints of the mice intra-articularly injected with *S. aureus* wild-type (WT) Newman and Newman Δ*lgt* strain EVs (**A**). Knee scale of the C57Bl/6 wild-type (WT) and toll-like receptor knockout (TLR2^−/−^) mice intra-articularly injected with *S. aureus* Newman EVs (**B**). Inflammation score of the synovitis tissue (**C**) and representative images of hematoxylin and eosin staining of knee joints of WT (**D**) and TLR2^−/−^ (**E**) mice. Knee scale of NMRI mice treated with Clodronate liposomes and PBS control liposomes intra-articularly injected with *S. aureus* Newman EVs (**F**). Statistical analyses were performed using the Mann–Whitney U test and the data represented as the mean with SEM. * *p* < 0.05; ** *p* < 0.01. Arrows in D indicate leukocyte infiltration.

Since *S. aureus* EVs induce cytokine expression in vitro, we further investigated whether these EVs are pro-inflammatory in vivo. EVs of Newman and Newman Δ*lgt* were intra-articularly (i.a.) injected into mouse knee joints. Clinical signs of arthritis were observed in the knee joints injected with Newman EVs after 24 h, and the knee sizes were significantly bigger compared to the healthy knees (Figure 5A). Interestingly, mice injected with Δ*lgt* exhibited less pronounced macroscopic arthritis than those injected with the parental strain, with similar sizes as the healthy controls (Figure 5A), suggesting the Lpps in *S. aureus* EVs are the culprits that induce inflammation in vivo, which is in line with the in vitro results.

To extend the above observations, Newman EVs were i.a. injected into the knee joints of TLR2^−/−^ and C57Bl/6 wild-type mice. Significantly less severe joint inflammation was observed in the knee joints of TLR2^−/−^ mice compared to the control mice at 24 h after injection (Figure 5B). Subsequently, we performed H&E staining to evaluate the differences in inflammation between wild-type and TLR2^−/−^ mice tested with Newman EVs. Leukocyte infiltration was apparent in the joints from C57Bl/6 wild-type mice i.a. injected with EVs (Figure 5D) with pronounced synovitis (Figure 5C) compared with TLR2^−/−^ mice, where there was no migration of cells (Figure 5E) with negligible inflammation (Figure 5C), suggesting that *S. aureus* EVs induce inflammatory reactions through TLR2-dependent mechanisms.

We recently showed that *S. aureus* Lpps induce arthritis mediated by monocytes/macrophages [28]. To further examine this potential link with the *S. aureus* EVs, mice with depleted monocytes/macrophages were i.a. injected with Newman EVs. There was a clear reduction in joint inflammation measured by knee size in monocyte/macrophage-depleted mice compared to the control mice (Figure 5F), implying that monocytes/macrophages also play a role in *S. aureus* EVs-induced inflammation.

## 3. Discussion

The study of bacteria cell-derived vesicles is a rapidly expanding and exciting new area. While significant progress has been made in the last decade, our understanding of the molecular mechanisms underlying vesicle release and their roles in infections is incompletely understood with *Staphylococcus aureus*. The key finding of the current study is that the Lpps was the main causative factor responsible for the pro-inflammatory properties of *S. aureus* EVs. This finding is novel but not very surprising, as Lpps are known to be the most potent bacterial component in *S. aureus* causing inflammation [28].

Lpps is one of the major types of surface proteins found in *S. aureus* [39]. Lpps play a role in a variety of surface-related functions, including feeding, respiration, protein secretion, and invasion [40,41]. Lpp consists of a protein moiety and a lipid moiety, which aids bacteria with metabolic nutrition and anchors the protein into the membrane, respectively [37,40]. The lipid moiety of *S. aureus* Lpps is known to be responsible for inducing strong immune responses via TLR2 [37]. Lpps expression is pathogenic in many *S. aureus* infections, such as sepsis [42], skin infection [43], and hematogenous septic arthritis [44], but is protective in local *S. aureus* septic arthritis [28]. Intriguingly, the Δ*lgt* strain defective in Lpp maturation released EVs with greater variation in sizes compared to its parental strain. Similarly, EVs from sortase mutants that lack major surface proteins also had a greater variation in size. We speculate that the greater variation in size of EVs may indicate the instability of the bacterial cell wall, and mature Lpps and surface proteins may participate in the process of EV production.

It has been shown that Lpps enrich *S. aureus* EVs [36]. The immune-stimulating capacity of EVs from the Δ*lgt* strain was greatly reduced compared with EVs from its parental strain, suggesting that Lpps are the main cause for the pro-inflammatory property of *S. aureus* EVs. However, a small increase in cytokine production was detectable when immune cells were stimulated with EVs from the Δ*lgt* strain compared with non-stimulated cells. This indicates that some other bacterial components, such as DNA [45] and peptidoglycan [46], in *S. aureus* also elicit a certain degree of the immune response, but negligible in comparison with Lpps. Surface proteins are a well-known major virulence factor of *S. aureus* and their virulence is mediated through adhesion to and invasion of host cells and tissues, as well as evasion of immune responses [47,48]. EVs from the Δ*srtAB* mutant fully preserved their capacities to induce immune responses, as surface proteins per se lack immunogenicity.

A subtle connection between lipoproteins and phenol-soluble modulins (PSMs) was initially observed by Peschel and colleagues. PSMα is largely required for mobilization of Lpps from *S. aureus*’s cytoplasmic membrane and consequent host TLR2 activation [49,50]. Additionally, PSMα destroys the cytoplasmic membrane and aids in the release of cytoplasmic proteins along with nucleic acids, lipids, and ATP [51]. Importantly, PSMα, but not PSMβ mutants, displayed a significantly reduced *S. aureus* EV yield [35], suggesting membrane damaging activity of PSMs may control the release of the EVs in *S. aureus*. TLR2 is known to be the dominant receptor of *S. aureus* Lpps. TLR2 deficiency deprives the responsiveness of immune cells to stimulation of *S. aureus* EVs. Mohammad et al. demonstrated that monocytes/macrophages were responsible for Lpps induced joint inflammation when Lpps were intra-articularly injected into mouse knees [28]. Indeed, in the current study mice depleted with monocytes/macrophages failed to develop joint inflammation when their knees were injected intra-articularly with *S. aureus* EVs. All these data further strengthen our conclusion that Lpps are the major pro-inflammatory components in *S. aureus* EVs.

Lipopolysaccharide (LPS), the major outer membrane component of Gram-negative bacteria, is one of the most potent bacteria-derived molecules activating the host immune response [52]. TLR4 is known to transduce LPS signals [52]. We speculate that in comparison with Lpp in *S. aureus*, LPS might be the main component in Gram-negative bacteria EVs in activating immune responses. Indeed, EVs isolated from Gram-negative bacteria such as *Escherichia coli* (*E. coli*) can regulate neutrophil migration [53] and induce lung emphysema via the IL-17 pathway [54]. TLR4 was found to recognize *E. coli* EVs and control the EVs-mediated IL-8 production in human endothelial cells [53]. Moreover, feces EVs containing both Gram-positive and Gram-negative bacterial EVs are capable of inducing sepsis-like systemic inflammation and this effect is mediated through both TLR2 and TLR4, respectively [55].

It is unclear how much *S. aureus* EVs contribute to *S. aureus-*mediated infections. The clinical signs of arthritis induced by *S. aureus* EVs were relatively mild despite the high concentration of EVs. At the same time, the yield of EVs by *S. aureus* was relatively low. However, the clinical significance of the pro-inflammatory effect of *S. aureus* EVs in disease pathogenesis cannot be ruled out, since Lpps are known to act in synergy with other bacterial components such as peptidoglycan to stimulate the immune response [56] and this synergistic effect may only require a minimal amount of Lpps. Genetically engineered *S. aureus* EVs expressing detoxified cytolysins were shown to be effective to prevent lethal sepsis in mice, paving the path to new vaccination strategies [35]. Our finding is important for the further development of such a vaccine. Immune-stimulatory effects of Lpps may induce more severe inflammatory responses, causing side effects, and it might be necessary to develop the EV vaccines using the Δ*lgt S. aureus* strain to eliminate the potential risk of side effects.

Interestingly, we observed that the average sizes of EVs from Δ*lgt* and Δ*srt A/B* strains were larger than EVs from the wild-type strain Newman (Figure 4A–C). It is a common phenomenon that the size of EVs is slightly larger than the cutoff filters (e.g., 0.2 micron filters) used in the isolation procedure, as EVs tend to bind to other particles, including other EVs. We presume that the EVs from Δ*lgt* and Δ*srt A/B* strains had a greater binding property to other proteins/particles/EVs.

**Figure 6:**
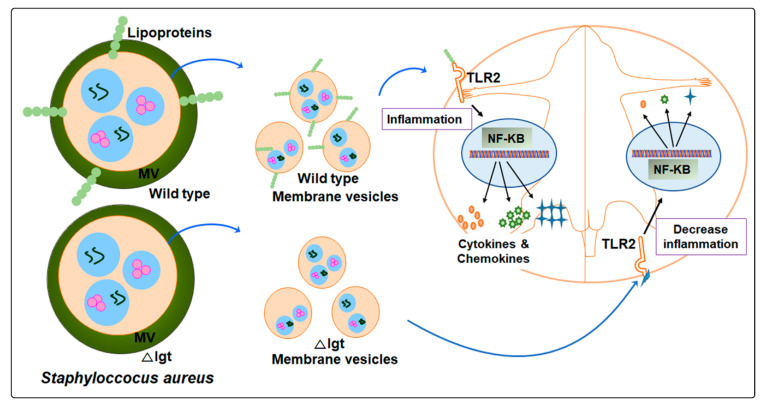
A proposed model explaining the role of *S. aureus* EVs lipoproteins in inducing inflammation through TLR2 signaling both in vitro and in vivo. *S. aureus* EVs contain proteins, nucleic acids, and lipoproteins. The wild-type Newman EVs harbor lipoproteins responsible for inducing inflammation in mice through TLR2, resulting in the release of a vast amount of chemokines and cytokines, whereas Δ*lgt* EVs, lacking lipoproteins, result in decreased inflammation with subsequent reduced production of chemokines and cytokines. 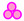
—Proteins; 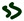
—Nucleic acids; MV: membrane vesicle.

Based on the overall data of this study, a schematic representation is shown in Figure 6. The Newman parental strain derived EVs, which contain nucleic acids and bacterial proteins including Lpps, caused inflammation in the host by activating the NF-kB pathway through TLR2 signaling and inducing the release of cytokines and chemokines, whereas *Δlgt* strain derived EVs almost totally lost their capacity to induce inflammation both in vitro and in vivo. In conclusion, to our knowledge, this is the first study demonstrating that Lpps are the major component responsible for the pro-inflammatory property of *S. aureus* EVs.

## 4. Materials and Methods

### 4.1. Bacterial Strains

*S. aureus* strains: Wild-type Newman [57]; Newman Δ*lgt* that is deficient in prelipoprotein lipidation [58], and Newman Δ*srtAB* that lacks Sortase A/B expression [59]. Newman strain was cultured with aeration in tryptic soy broth (TSB) at 37 °C for 48 h, while the mutant strains, Newman Δ*lgt* and Newman Δ*srtAB*, were cultured in TSB with erythromycin (2.5 µg/mL) for 48 h at 37 °C.

### 4.2. Ethics

All of the experiments were performed according to the guidelines of the Swedish Research Council and local ethical guidelines from recommendations listed in the Swedish Board of Agriculture’s regulations and recommendations on animal experiments. The experiments are approved by the regional animal ethics committee (Jordbruksverket) of the University of Gothenburg with Dnr: 38-2016 (approved date 27042016 and valid until 26042021).

### 4.3. Mice

Female NMRI and C57Bl/6 wild-type mice of both sexes, aged 6–9 weeks, were purchased from Envigo (Venray, The Netherlands) and Charles River Laboratories (Sulzfeld, Germany), respectively. Toll-like receptor 2-deficient B6.129-Tlr2tm1Kir/J (TLR2^−/−^) mice of both sexes were purchased from The Jackson Laboratory (Bar Harbor, ME, USA). All mice were housed in the animal facility of the Department of Rheumatology and Inflammation Research, University of Gothenburg. Mice were kept under standard temperature and light conditions and were fed laboratory chow and water ad libitum.

### 4.4. Isolation of EVs

*S. aureus* was cultured with approx. 0.5–1 L of TSB via shaking for 48 h. The culture was centrifuged at 5000× *g* rpm for 10 min. The supernatant was centrifuged again for 30 min at 5000× *g* rpm. Furthermore, the supernatant was ultracentrifuged in an Optima L-100 XP ultracentrifuge with 70Ti fixed-angle rotor (Beckman Coulter) by using Beckman centrifuge tubes for 30 min at 19,400× *g* rpm. The resultant supernatant was filtered using 0.2 µm Millipore filters and centrifuged again for a further 90 min at 38,800× *g* rpm. The EV pellet was washed and dissolved with filtered (0.1 µm) PBS and stored at −20 °C. After isolation of EVs, 1/6th portion of the EVs was used to check for sterility on a horse blood agar plate. The protein concentration of EVs was measured using Qubit 2.0 (Invitrogen, Carlsbad, CA, USA). EVs’ size distribution and particle diameter were measured using a NanoSight LM10 (Malvern Panalytical), according to previously established analysis settings [60].

### 4.5. Electron Microscopy

#### 4.5.1. SEM Sample Preparation

Forty-eight 5 × 5 mm Si wafers (Micro to Nano 10-008145) were coated with 0.1% aq poly-L-lysine (Sigma P8920) for 10 min and air-dried. The wafers were placed in 24 well plates (two wafers per well) and bacterial culture was added (1ml per well) for 40 min for the cells to adhere. The culture was removed and RT Karnovsky fixative was immediately added (1 mL per well) for overnight incubation at 4 °C. The sample was washed 5× with 0.1 M cacodylate buffer for a total of 1h and postfixed using the OTO method (1% OsO_4_ in 0.1 M cacodylate buffer for 30 min, washing several times with water, 1% aqTCH for 20 min, 1% aq OsO_4_ for 45 min), dehydrated in EtOH (30%, 50%, 70%, 85%, 95%, 3× 100%, 5 min per step), and dried using the HMDS method (incubation in HMDS for 2 min, removal of the HMDS, and air-drying). The samples were coated with 3 nm Au using a Quorum Q150T sputter coater. The samples were imaged using Zeiss Gemini 450 II SEM.

#### 4.5.2. Negative Staining for TEM Imaging

In-house-made carbon-formvar grids (Cu 150 mesh) were glow discharged using Quorum GloQubePlus (15 mA for 40 s, positive source polarity). They were immediately placed support film side down on a 20 μL drop of vesicle suspension (0.25 µg/mL), incubated for 5 min, blotted vertically against filter paper, moved to a 20 μL droplet of 2% aq uranyl acetate for 10 s, blotted and air-dried. The grids were imaged using Talos L120C TEM at 120 kV.

### 4.6. In Vitro Peritoneal Macrophages and Splenocytes Stimulation

Peritoneal macrophages and splenocytes were extracted from healthy NMRI, C57Bl/6, and TLR2^−/−^ mice under sterile conditions, as previously described [28]. Briefly, peritoneal macrophages were collected with 10 mL of ice-cold PBS at the mouse peritoneal region. The cells were treated with 0.83% ammonium chloride if RBC cells were present and the remainder were washed with PBS. The washed cell pellets were suspended in Iscove’s complete medium (10% fetal calf serum, 2 mM L-glutamine, 5 × 10^−5^ M mercaptoethanol, and 50 μg/mL gentamicin) to a density of 1 × 10^6^ cells/mL.

For the splenocyte culture, the mouse spleens were removed, mashed aseptically, and passed through a 70 µm cell strainer (Fisher Scientific, Waltham, MA, USA). The RBC cells were lysed with 0.83% ammonium chloride and the cells were washed before suspending in Iscove’s complete medium to a density of 2 × 10^6^ cells/mL.

30 ng/µL and 3 ng/µL of purified EVs were used for the stimulation of peritoneal macrophages and splenocytes. Purified Lpl1 [61] (0.2 µg/mL) and lipopolysaccharide (LPS) (1 µg/mL) were used as positive controls while Iscove’s media were used as negative controls. Peritoneal macrophages and splenocytes were incubated (37 °C, 5% CO_2_) for up to 24 and 48 h, respectively. The supernatants were collected at 4 and 24 h for peritoneal macrophages, and 24 and 48 h for splenocytes, for the cytokine/chemokine analysis.

### 4.7. In Vivo Experiments

The mouse model for bacterial components induced arthritis was adapted from a previous study [62]. 20 µL (1 µg/µL) of Newman and Δ*lgt S. aureus* EVs were intra-articularly (i.a.) injected into five NMRI mice on both knees. After 24 h the knee joints were measured, isolated in sterile conditions, and homogenized with 600 µL of PBS. The resultant supernatant was stored at −20 °C for further analysis.

In another set, 20 μL (0.7 µg/µL) of Newman EVs were i.a. injected into both knee joints of C57Bl/6 wild-type (*n* = 6,) and TLR2^−/−^ (*n* = 6) mice. After 24 h the knee joints were measured, isolated, and stored in 4% formaldehyde for hematoxylin and eosin (H&E) staining.

### 4.8. In Vivo Macrophage Depletion

Clodronate liposomes (Liposoma BV, Amsterdam, The Netherlands) or PBS control liposomes were injected into NMRI mice (*n* = 6 mice; clodronate liposomes *n* = 6 joints; PBS control liposomes *n* = 6 joints) both on the knee (i.a. 20 µL/joint) and tail (i.v. 200 µL/mouse) for eliminating monocytes/macrophages [63] one day before testing with Newman EVs (20 µg in 20 µL).

### 4.9. Enzyme-Linked Immunosorbent Assay (ELISA)

The levels of Macrophage-Inflammatory Protein 2 (MIP-2), tumor necrosis factor (TNF-α), and interleukin 6 (IL-6) in the supernatants of peritoneal macrophages and splenocytes stimulation were detected with DuoSet ELISA Kits (R&D Systems, Abingdon, UK) according to the manufacturer’s instructions.

### 4.10. H&E Staining

The mouse knee joints were fixed in formalin for at least 72 h before proceeding with decalcification. Later the joints were grossed to remove excess tissue, enclosed in respectively coded cassettes, and submerged in decalcification solution (10% EDTA in 0.1 M Tris-buffer, adjust pH with KOH pellets to 6.95) for 2 weeks with intermittent changes of solution every 2 days. After decalcification, joints were embedded in paraffin and 4 µm sections were cut using a microtome (Leica DMR, Wetzlar, Germany). Tissue sections were thereafter stained for hematoxylin and eosin with an automatic staining machine (LinearStain II Tissue-Tek, Sakura Fintek, Torrance, CA, USA). All slides were coded and assessed under a microscope (Leica DMR, Wetzlar, Germany) in a blinded manner. The extent of inflammation in the synovial region was judged.

### 4.11. Statistical Analysis

All statistical analyses were performed using GraphPad Prism version 9 (GraphPad Software, La Jolla, CA, USA). Statistical differences were assessed using the Mann–Whitney U test and data represented as the mean with standard error of the mean (SEM). * *p* < 0.05; ** *p* < 0.01; *** *p* < 0.001; **** *p* < 0.0001.

## Data Availability

The data presented in this study are available on request from the corresponding author.

## Supplementary Materials

The following are available online at https://www.mdpi.com/article/10.3390/ijms22137099/s1.
