# Supplementary material for: Lipoproteins Are Responsible for the Pro-Inflammatory Property of Staphylococcus aureus Extracellular Vesicles
Source: Int J Mol Sci. 2021 Jul 1;22(13):7099. doi: 10.3390/ijms22137099 (PMC8268867; doi:10.3390/ijms22137099)
Supplement: Supplementary file 1 [file ijms-22-07099-s001.zip › ijms-1276125-supplementary.pdf]

Supplementary Materials

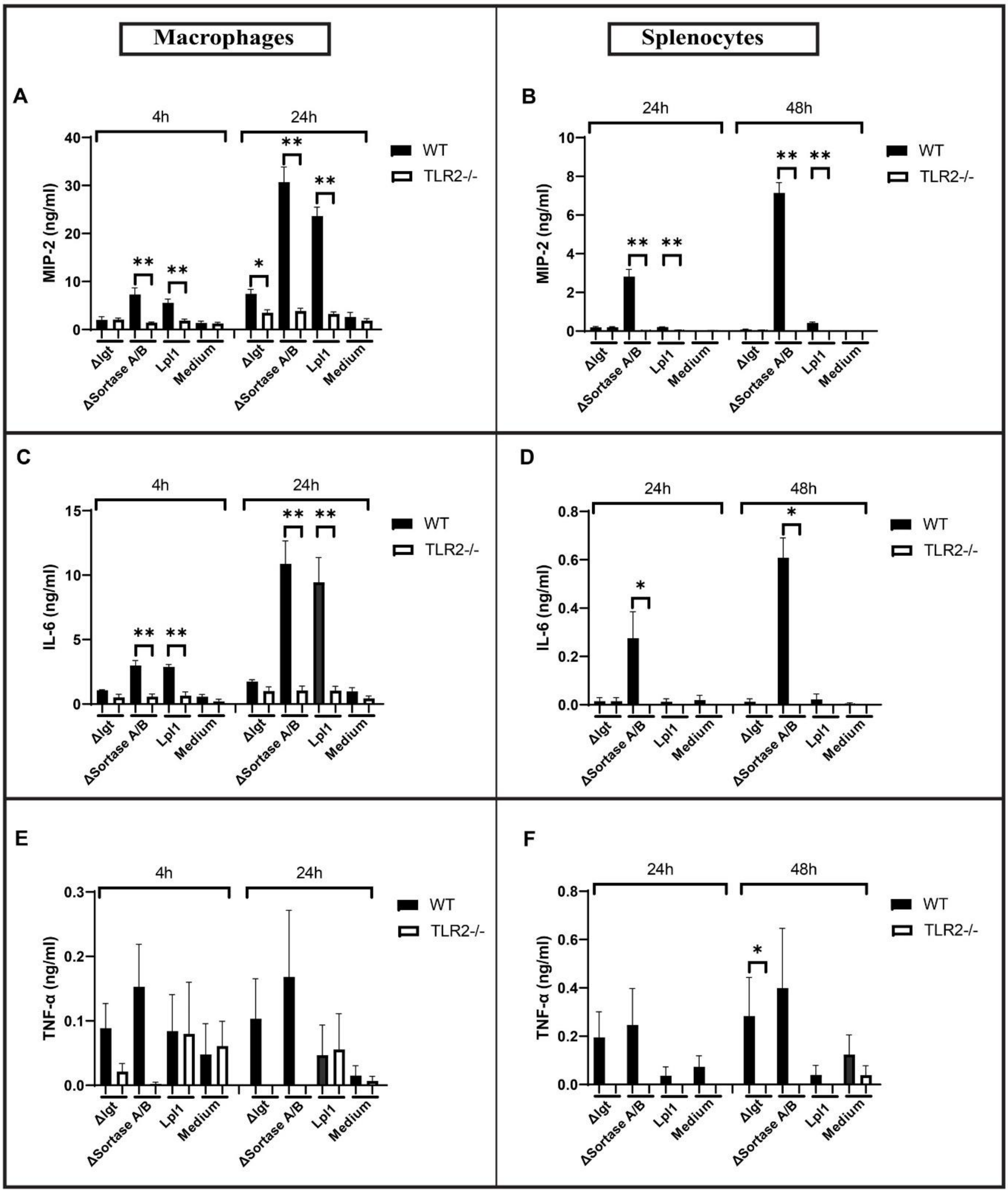

Supplementary Figure S1. *In vitro* stimulation of *S. aureus*, Δ*lgt* and Δ*srtAB* derived EVs in murine peritoneal macrophages and splenocytes of C57Bl/6 wild-type (WT) and Toll-like receptor 2 deficient mice (TLR2$^{-/-}$). A, C, and E: MIP2, IL-6, and TNF-α stimulation in peritoneal macrophages while B, D, and F: MIP2, IL-6, and TNF-α stimulation in splenocytes. Statistical analyses were performed using the Mann-Whitney U test and the data represented as the mean with SEM * P<0.05; ** P<0.01.

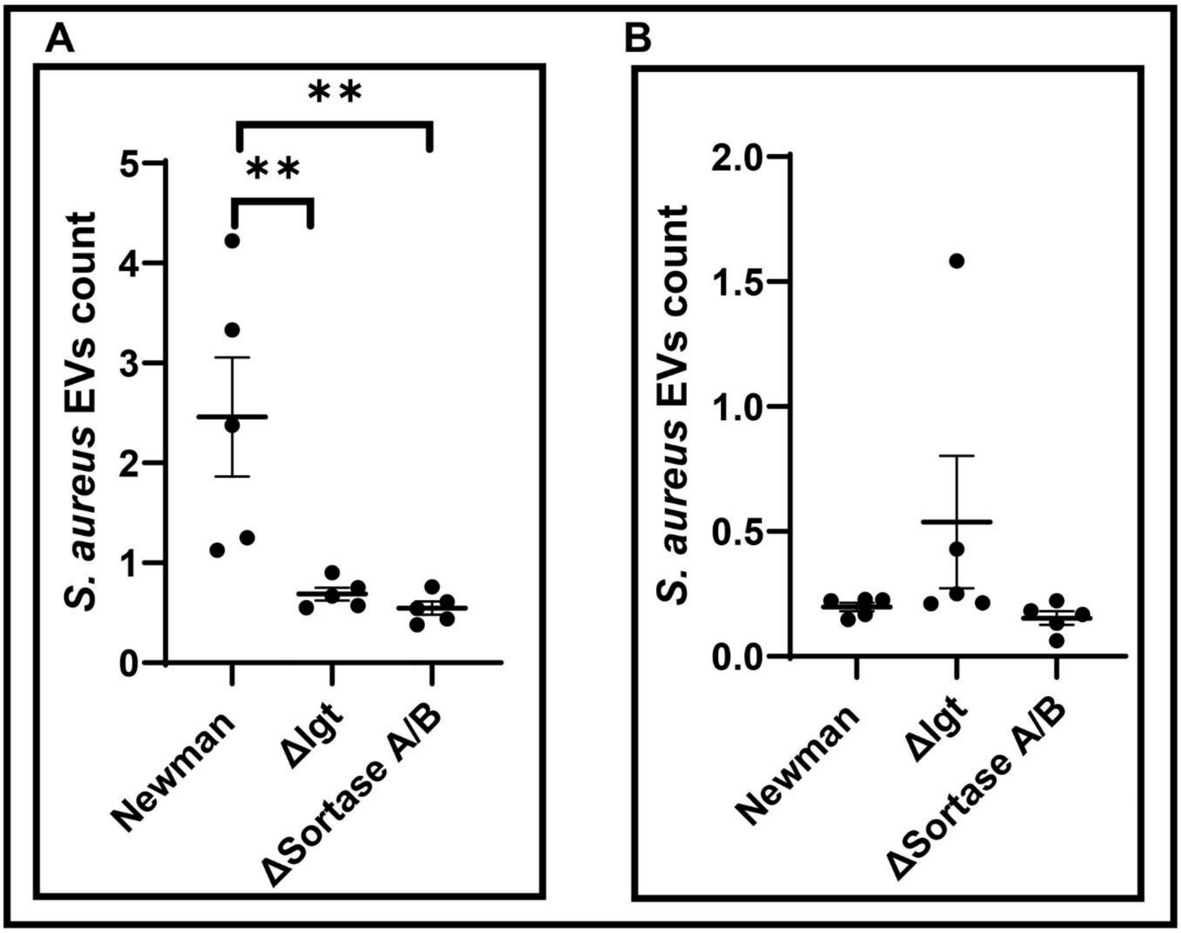

Supplementary Figure S2. Quantification of EVs in bacterial culture. **A**: 3h bacterial culture, **B**: 24h bacterial culture. Statistical analyses were performed using the Mann-Whitney U test, and the data represented as the minimum to maximum with * P<0.05; ** P<0.01.

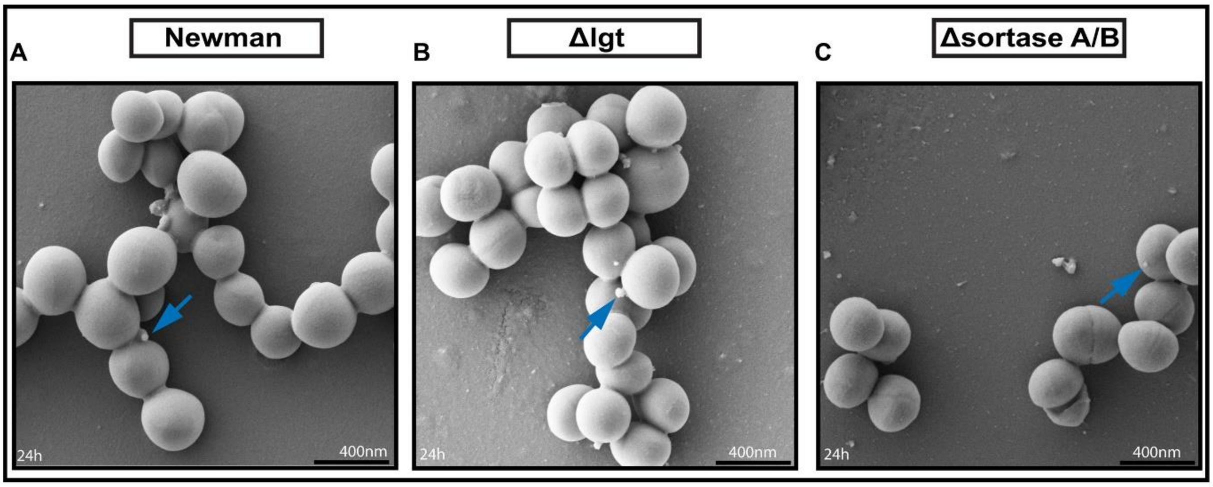

Supplementary Figure S3. Release of EVs from *S. aureus* cultures after 24h. Parental strain Newman (**A**), Δ*lgt* mutant (**B**), and Δ*srtAB* mutant (**C**). Budding like dots on the cell surface of *S. aureus* can be seen in the A, B, and C images, indicating EVs.
